# Involvement of the GABAergic and the serotonergic systems in the anxiolytic effects expressed by the nitric oxide (NO) donor sodium nitroprusside (SNP) in the male rat

**DOI:** 10.1007/s00213-025-06759-1

**Published:** 2025-02-18

**Authors:** Maria Anastasia Parlantza, Nikolaos Pitsikas

**Affiliations:** Department of Pharmacology, School of Medicine, Faculty of Health Sciences, University of Thessaly, Panepistimiou 3, Biopolis, Larissa, 415-00 Greece

**Keywords:** Nitric oxide, Sodium nitroprusside, Flumazenil, 8-OH-DPAT, Anxiety

## Abstract

**Rationale:**

Anxiety is a chronic severe psychiatric disorder. In a series of studies, the implication of the gaseous molecule nitric oxide (NO) in anxiety has been evidenced. Further, the outcome of preclinical research suggests that different NO donors, including sodium nitroprusside (SNP), have expressed an anxiolytic profile revealed in animal models of anxiety. Regardless of this, it is not yet clarified the mechanism(s) of action by which SNP induces its beneficial effects on anxiety. In this context, it has been hypothesized that these effects might be attributed to a potential interaction of this NO donor with the GABA type A and the 5-HT_1A_ serotonergic receptors.

**Objectives:**

The current study was designed to investigate this issue in the male rat.

**Methods:**

To this end, the light/dark box and the open field tests were utilized.

**Results:**

SNP (1 mg/kg, i.p.) applied acutely induced an anti-anxiety-like effect evidenced either in the light/dark box or in the open field test. Either the GABA_A_ receptor antagonist flumazenil (10 mg/kg, i.p.) or the 5-HT_1A_ serotonin receptor agonist 8-OH-DPAT (0.25 mg/kg, i.p.) suppressed the above reported anxiolytic effects of SNP.

**Conclusions:**

The results here reported propose a functional interaction between SNP with the GABAergic and the serotonergic systems on anxiety and thus, might offer a plausible explanation for SNP’s anxiolytic effects.

## Introduction

Anxiety is a severe psychiatric disorder. Different subtypes of this psychiatric disorder, like generalized anxiety disorder (GAD), specific phobias (social phobia, claustrophobia, agoraphobia, etc.), post-traumatic stress disorder (PTSD) and panic disorder have been reported. Standard characteristics of all these disorders are provisional worry and excessive fear (Steimer 2018).

Even though a consistent number of chemicals belonging to different pharmacological classes are currently utilized to relief the symptoms of anxiety disorders [e.g., benzodiazepines, partial agonists of the serotonergic 5-HT_1A_ receptor, selective serotonin reuptake inhibitors (SSRIs)] different forms of anxiety, including panic, PTSD and social phobia, are not sensitive to these medications. (Hammer et al. 2004; Holt and Bruce Lydiard 2007; Van Ameringen et al. 2004). Moreover, these medications are frequently associated to undesired side effects (Cryan and Sweeney 2011).

Having stated this, there is an urgent requirement to discover and develop novel molecules for the treatment of the different forms of anxiety (Gorman 2003). Between the various strategies aimed at discovering new anxiolytic compounds, the implication of the donors of the gaseous molecule nitric oxide (NO) in this regard has been suggested.

NO is an important intra- and inter-cellular messenger in the brain (Dawson and Snyder 1994; Garthwaite 1991). The connection of NO in anxiety has been proposed although its precise role in this pathology has not yet been completely clarified (for review see Guimaraes et al. 2005; Pitsikas 2018). Sodium nitroprusside (SNP) is a NO donor with very short half-life (4 min) expressing potent consistent vasodilatory and anti-hypertensive properties (Kreye and Reske 1982; Varner and Beckman 1995). Further, SNP is currently under clinical evaluation as a potential novel neuroleptic (Hallak et al. 2013). Further, it is well documented that anxiety disorders are a typical feature in schizophrenia patients (for review see Braga et al. 2013).

In a series of preclinical behavioural studies, the anti-anxiety-like actions of SNP have been evidenced. Specifically, a single intraperitoneal injection of SNP (1 mg/kg) induced an anxiolytic effect revealed in the light/dark box test (Orfanidou et al. 2017) and in the contextual fear conditioning test (Peristeri and Pitsikas 2022). Further, repeated intraperitoneal application of this NO donor (0.1 and 0.3 mg/kg, for 5 consecutive days) induced an anxiolytic-like behaviour observed in the light/dark box and open field tests (Papageorgoulis et al. 2020). Moreover, the above reported anxiolytic effects of SNP seem to be related to the concentration and to the time between administration and testing (Papageorgoulis et al. 2020; Pitsikas 2018). The mechanism(s) of action through which SNP exerts its anti-anxiety-like effects, however, is not yet clarified.

It is clearly established that the GABA_A_ receptor is the target site of the anxiolytic action of the benzodiazepines (Shekhar 1993) whereas the anxiolytic effects of buspirone are attributed to its interaction with the 5-HT_1A_ receptor (Lucki and Wieland 1990). As such, it has been shown that SNP directly activates GABA_A_ receptor function (Castel and Vaudry 2001). Additionally, impediment of the NOergic activity has as consequence the reduction of the anxiolytic effects of benzodiazepines. In this regard, it has been observed that the NO synthase (NOS) inhibitor L-N^G^-nitro arginine (L-NOARG) antagonized the anxiolytic effect induced by chlordiazepoxide in the elevated plus maze test in mice (Elfline et al. 2004).

Immunohistochemical studies have demonstrated the co-existence of neuronal NOS (nNOS) and serotonin in the rat hippocampus, medial septum and amygdala, which are brain areas rich of 5-HT_1A_ receptors and critically involved in anxiety (Bredt et al. 1991; Dawson et al. 1991). Additionally, different behavioural findings propose an effective interplay between the 5-HT_1A_ receptor and the NOergic system which may be of relevance in aggressive behaviour and cognition. It has been shown that excessive and impulsive traits of the enzyme nNOS knockout (nNOS -/-) mice were caused by reduction of serotonin turnover and these increased aggressive brhaviour was attenuated by treatment with 5-HT_1A_ and 5-HT_1B_ receptor agonists (Chiavegatto and Nelson 2003). Moreover, the NO donor molsidomine was found able to counteract recognition memory impairments induced by the 5-HT_1A_ receptor agonist 8-hydroxy-2-(dipropylamino)tetralin (8-OH-DPAT) in the rat (Pitsikas et al. 2005).

Finally, the ability of NO donors to modulate, dose-dependently, GABA and serotonin release from different brain structures is also reported (Prast and Philippu 2001).

Taking into consideration the above reported information, the current study was designed to evaluate whether the GABA_A_ and/or the 5-HT_1A_ receptors might be potential targets of the anti-anxiety-like behaviour expressed by the NO donor SNP. Thus, the anxiolytic-like effects of SNP were challenged with the benzodiazepine receptor antagonist flumazenil and the 5-HT_1A_ receptor agonist 8-OH-DPAT. The light/dark box and the open field tests were the behavioural procedures utilized for this assessment (Crawley and Goodwin 1980; Prut and Belzung 2003). Finally, locomotor activity was also evaluated as an independent parameter of the potential motoric effects of the chemicals that could influence rodents’ performance in the light-dark box and the open field tests.

## Materials and methods

### Subjects

Independent groups of naïve male 3-month-old albino Wistar rats (188 animals) (Hellenic Pasteur Institute, Athens, Greece) that weighed 250–300 g were used in this study. The animals were housed in Makrolon cages (47.5 cm length x 20.5 cm height x 27 cm width), three per cage, in a climate-regulated environment (21 ± 1 °C; 50–55% relative humidity; 12-h/12-h light/dark cycle, lights on at 07.00 h) with free access to standard laboratory diet (pellets) for rats and water.

The procedures that involved animals and their care were conducted in accordance with international guidelines and national (Animal Act, P.D. 160/91) and international laws and policies (EU Directive 2010/63). Experiments were approved by the local committee (Prefecture of Larissa, Greece, protocol number 58379/2023). Every effort was made to minimize the number of animals used and their suffering.

### Behaviour

#### Light/dark (L/D) test

L/D test is a behavioural tool valid for assessing the anxiolytic- and anxiogenic-like effects of drugs in rodents. It is a quick and easy procedure; it can be used without previous practice and food, or water deprivation is not needed (Crawley and Goodwin 1980). Transitions in this procedure reflect activity/exploration because habituation over time is seen with this measure, while the time spent in each chamber of the apparatus resembles aversion/attraction (Belzung et al. 1987).

The L/D box apparatus consisted of a wooden box (48 cm length x 24 cm height x 27 cm width) divided into two equal-size chambers by a barrier that contained a doorway (10 cm height x 10 cm width). One of the chambers was painted black and was covered with a lid and the other compartment was painted white and illuminated with a 60-W light bulb positioned 40 cm above the upper edge of the box. The test was performed as described previously (Grivas et al. 2013). Each animal was placed in the middle of the lit compartment, facing away from the dark chamber. The rat was allowed to freely explore the apparatus for 5 min. The latency to enter (with all four paws) the dark compartment, number of transitions and time spent in the light and dark compartments were recorded.

#### Locomotor activity test

Locomotor activity was recorded to check for direct effects of chemicals on physical activity that could confound the interpretation of results the light-dark test.

Spontaneous motor activity was assessed in an activity cage (catalog number 7420, Ugo Basile, Varese, Italy). The apparatus consisted of a box made of Plexiglas (41 cm length x 33 cm height x 41 cm width). Every movement of the animal produced a signal caused by variations in the inductance and capacitance of resonance circuitry of the apparatus. The signals were then automatically converted into numbers that reflected horizontal activity counts. Changes in activity counts represent a standard behavioral assay for testing the motoric effects of drugs. The test was performed as described previously (Grivas et al. 2013). Each animal was placed into the locomotor activity arena and spontaneous locomotion was recorded for 5 min.

#### Open field (OF) test

OF test is a standard anxiety procedure evaluating neophobic behaviour in rodents. In this test, animals routinely tend to elude open spaces. The amount of time spent by rodents in the central zone of an open field apparatus, therefore, is an estimation of an anxiety state (Prut and Belzung 2003).

The test apparatus consisted of a dark open box made of Plexiglas (70 cm length x 50 cm height x 70 cm width). The open field arena was divided-by black lines-into 16 squares of 17.5 × 17.5 cm^2^. The central four squares were defined as the central zone, in which animals’ activity was regarded as a measure of anxiety (Prut and Belzung 2003). The test was performed as described previously (Grivas et al. 2013; Kalouda and Pitsikas 2015). Each animal was then placed in the same corner of the open field arena and its behaviour was recorded for 5 min. The variables observed were: (a) the amount of the time spent in the central zone of the open field arena as defined by all forepaws being in the central four squares of the apparatus, (b) the number of squares crossed (i.e., horizontal activity), and (c) the number of rearing behaviours (i.e., vertical activity, defined as raising both forepaws above the floor while balancing on hind limbs).

### Drugs

SNP (Merck KGaA, Darmstadt, Germany) was dissolved in saline (NaCl 0.9%) and protected from light to prevent photodecomposition until injection (Bisset et al. 1981). The dose of SNP (1 mg/kg) which produced the highest anxiolytic effect was selected based on the results of a previous study (Orfanidou et al. 2017). Flumazenil (Sigma, St. Louis, MO, USA) was dissolved in saline containing 0.1% Tween 80. Flumazenil dose (10 mg/kg) was chosen based on previous studies (Pitsikas and Tarantilis 2020; Schmidt-Mutter et al. 1998) in which this dose was found to abolish the anti-anxiety effects of various potential anxiolytic agents. 8-OH-DPAT (Sigma, St. Louis, MO, USA) was dissolved in saline. The 8-OH-DPAT dose (0.25 mg/kg) was chosen based on a prior report in which it has been demonstrated that this dose exerted an anxiogenic effect in rats evidenced in the light/dark test (Arran et al. 2013). All compounds were prepared on the day of the test and were administered intraperitoneally (i.p.) in a volume of 1 ml/kg. For all studies, control animals received isovolumetric amounts of the specific vehicle solution used in each study (NaCl 0.9%).

### Experimental protocol

Experiments were conducted between 10:00 and 14:00 during the light phase of the light/dark cycle. Different groups of rats were utilized across different studies. Each rat was tested only once. On the testing day, the animals were transported to the test darkened room and left undisturbed in their home cages for 2 h. To avoid the presence of olfactory cues, all the apparatuses (light/dark box, motor activity cage and open field arena) were thoroughly cleaned with 20% ethanol and then wiped with dry paper after each trial. Behaviour was video recorded. Data evaluation of experiments 1, 3, 4 and 6 was subsequently performed using a stopwatch by an experimenter who was unaware of the pharmacological treatment of each subject. Motor activity data from experiments 2 and 5 was automatically provided by the test apparatus.

### Experiment 1: effects of acute administration of SNP and flumazenil on rats’ performance in the L/D box test

Animals were randomly divided into four experimental groups with 9 rats per group as follows: vehicle (NaCl 0.9%) + vehicle (NaCl 0.9% containing 0.1% Tween 80), vehicle (NaCl 0.9% containing 0.1% Tween 80) + flumazenil (10 mg/kg), vehicle (NaCl 0.9%) + SNP (1 mg/kg), and flumazenil (10 mg/kg) + SNP (1 mg/kg). Compounds were administered 30 min before testing.

### Experiment 2: effects of acute administration of SNP and flumazenil on rats’ performance in the motor activity test

Animals were randomly divided into four experimental groups with 6 rats per group. The same experimental design used in experiment 1 was applied in experiment 2.

### Experiment 3: effects of acute administration of SNP and flumazenil on rats’ performance in the OF test

Animals were randomly divided into four experimental groups with 8 rats per group. The experimental design used in experiment 1 was applied in experiment 3.

### Experiment 4: effects of acute administration of SNP and 8-OH-DPAT on rats’ performance in the L/D box test

Animals were randomly divided into four experimental groups with 8 rats per group as follows: vehicle (NaCl 0.9%) + vehicle (NaCl 0.9%), vehicle (NaCl 0.9%) + 8-OH-DPAT (0.25 mg/kg), vehicle (NaCl 0.9%) + SNP (1 mg/kg), and 8-OH-DPAT (0.25 mg/kg) + SNP (1 mg/kg). SNP and 8-OH-DPAT were administered 30 and 10 min respectively before testing.

### Experiment 5: effects of acute administration of SNP and 8-OH-DPAT on rats’ performance in the motor activity test

Animals were randomly divided into four experimental groups with 8 rats per group. The experimental design used in experiment 4 was applied in experiment 5.

### Experiment 6: effects of acute administration of SNP and 8-OH-DPAT on rats’ performance in the OF test

Animals were randomly divided into four experimental groups with 8 rats per group. The experimental design used in experiment 4 was applied in experiment 6.

### Statistical analysis

Data are expressed as mean ± SEM. Data were analyzed utilizing two-way analysis of variance (ANOVA) test. The two-way ANOVA compares the mean differences between groups that have been split on two independent variables (called factors). The factors were SNP and flumazenil (experiments 1–3) or SNP and 8-OH-DPAT (experiments 4–6). The primary purpose of a two-way ANOVA is to understand if there is an interaction between the two independent factors on the dependent variable test (light/dark, motility, open field). Post-hoc comparisons between treatment means were made using Tukey’s *t* test, but only when a significant interaction was achieved. Values of *p* < 0.05 were considered statistically significant (Kirk 1968).

## Results

### Experiment 1: effects of acute administration of SNP and flumazenil on rats’ performance in the L/D box test

**Fig. 1:**
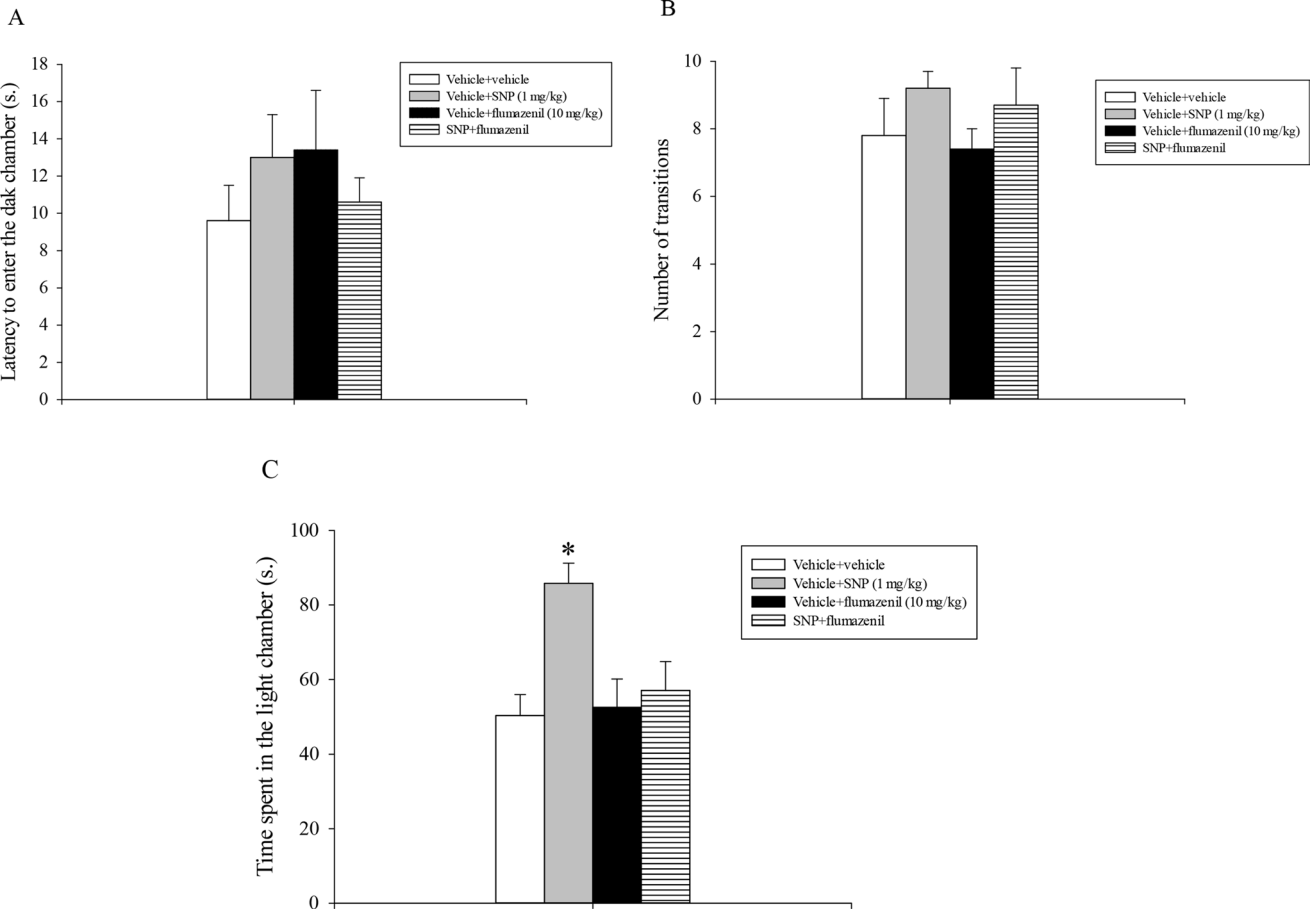
(**A**). Light/dark box test. SNP and flumazenil were injected i.p. 30 min before testing. The histogram shows the means ± SEM of 9 rats per treatment group. (**A**) Latency to enter the dark chamber. (**B**) Number of transitions. (**C**) Time spent in the light chamber. *p < 0.05 vs. all the other groups

Analysis of the first entry into the dark chamber (Fig. 1A) and the number of transitions between the two compartments (Fig. 1B) data did not reveal a statistically significant main effect of both flumazenil and SNP or a statistically significant interaction of flumazenil and SNP. Analysis of the total time spent by rats in the aversive light chamber (Fig. 1C) showed a statistically significant main effect of flumazenil (*F*_1,35_ = 8.98, *p* < 0.001) of SNP (*F*_1,35_ = 3.98, *p* = 0.05) and a statistically significant interaction between flumazenil and SNP (*F*_1,35_ = 5.35, *p* = 0.027).

Post-hoc analysis conducted subsequently demonstrated that animals that received vehicle + SNP (1 mg/kg) spent more time in the light chamber of the apparatus compared to all the remaining experimental groups (*p* < 0.05, Fig. 1C).

### Experiment 2: effects of acute administration of SNP and flumazenil on rats’ performance in the motor activity test

**Fig. 2:**
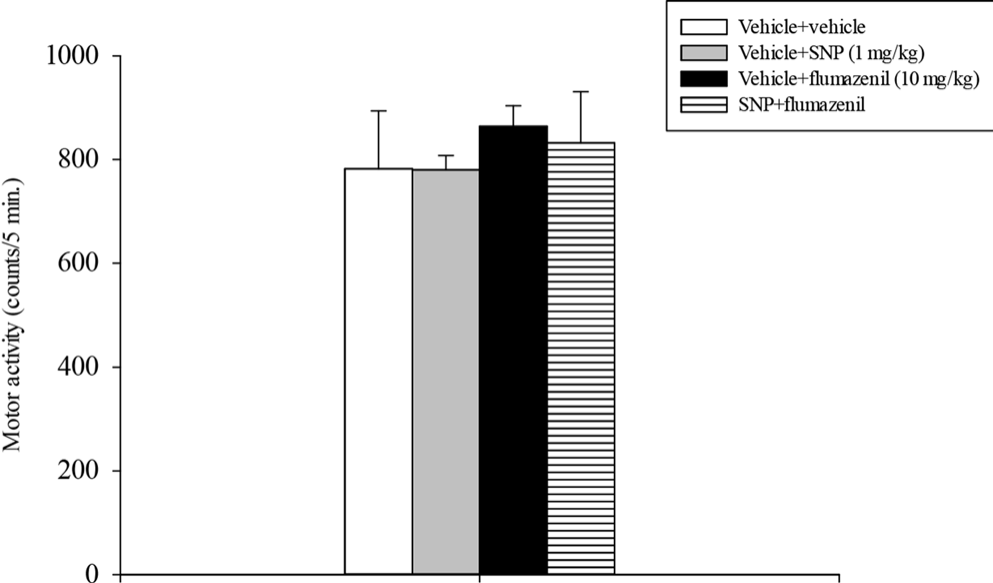
Locomotor activity test. SNP and flumazenil were injected i.p. 30 min before testing. The histogram shows the means ± SEM of 6 rats per treatment group

Motor activity data analysis did not evidence any effect either of flumazenil or of SNP or a statistically significant flumazenil x SNP interaction (Fig. 2).

### Experiment 3: effects of acute administration of SNP and flumazenil on rats’ performance in the OF test

Relative results are illustrated in Table 1. The number of squares crossed and number of rearings were not affected either by flumazenil or SNP. Regarding the total amount of time spent by rats in the central area of the open field, a statistically significant interaction between flumazenil and SNP was revealed (*F*_1,31_ = 10.39, *p* = 0.003). Further, a significant main effect of SNP (*F*_1,31_ = 21.98, *p* < 0.001) but not of flumazenil was evidenced.

**Table 1:** Effects of acute treatment with sodium nitroprusside (SNP) and flumazenil on rats’ performance in the open field test

TREATMENT	Number of squares crossed	Number of rearings	Time spent in the central zone (s.)
Vehicle + vehicle	65.9 ± 2.4	32 ± 2.5	3.5 ± 1
SNP (1 mg/kg) + vehicle	76.5 ± 8.1	34.1 ± 2.1	17.3 ± 3.9*
Flumazenil (10 mg/kg) + vehicle	80.5 ± 7.3	34.9 ± 1.9	6.9 ± 1.6
Flumazenil + SNP	84 ± 5.4	39 ± 1.7	7.5 ± 2.5

SNP and flumazenil were injected i.p. 30 min before testing respectively

The values are mean ± SEM (*n* = 8 rats per group). *p < 0.05 vs. all the other groups

Post-hoc analysis carried out showed that animals that have received vehicle + SNP spent more time in the central zone of the open field arena as compared to the other counterparts (*p* < 0.05).

### Experiment 4: effects of acute administration of SNP and 8-OH-DPAT on rats’ performance in the light/dark test

**Fig. 3:**
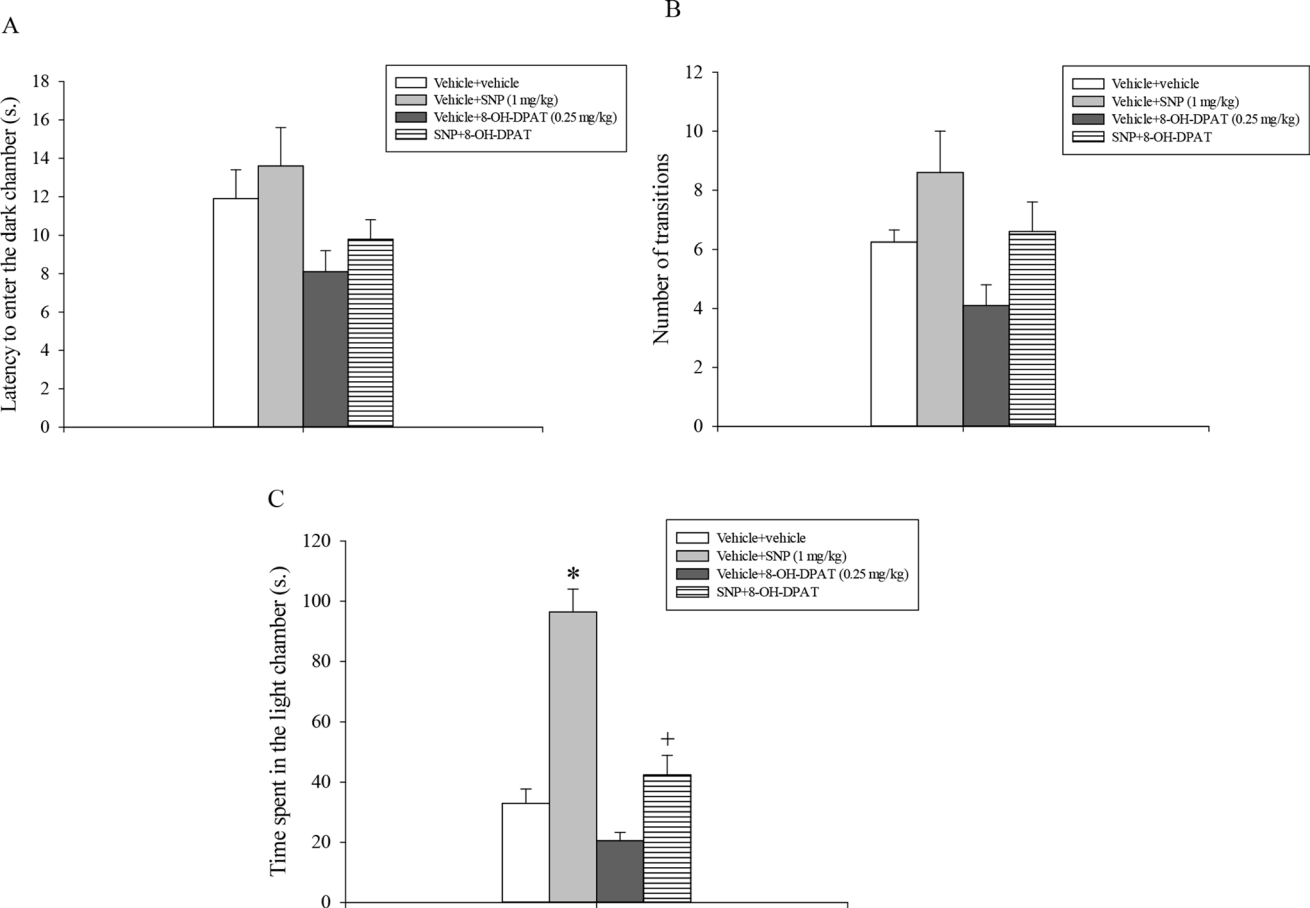
(**A**). Light/dark box test. SNP and 8-OH-DPAT were injected i.p. 30 and 10 min respectively before testing. The histogram shows the means ± SEM of 8 rats per treatment group. (**A**) Latency to enter the dark chamber. (**B**) Number of transitions. (**C**) Time spent in the light chamber. *p < 0.05 vs. all the other groups

Analysis of the first entry into the dark chamber showed a statistically significant main effect of 8-OH-DPAT (*F*_1,31_ = 6.88, *p* = 0.014) but not of SNP or a statistically significant SNP x 8-OH-DPAT interaction (Fig. 3A). Analysis of the data related to the number of transitions between the two compartments revealed a significant main effect of 8-OH-DPAT (*F*_1,31_ = 4.85, *p* = 0.036), of SNP (*F*_1,31_ = 6.78, *p* = 0.015) but not a significant interaction between 8-OH-DPAT and SNP (Fig. 3B). Analysis of the total time spent by rats in the aversive light chamber (Fig. 3C) showed a statistically significant main effect of 8-OH-DPAT (*F*_1,31_ =34.03, *p* < 0.001) of SNP (*F*_1,31_ = 56.31, *p* < 0.001) and a statistically significant interaction between 8-OH-DPAT and SNP (*F*_1,31_ = 13.38, *p* < 0.001).

Post-hoc analysis conducted subsequently on these data demonstrated that animals that received vehicle + SNP (1 mg/kg) spent significantly more time in the light chamber of the apparatus as compared to all the remaining experimental groups. In addition, the group of rats treated with SNP + 8-OH-DPAT remained more time in the central zone of the OF as compared to the vehicle + vehicle treated rats (*p* < 0.05, Fig. 3C).

### Experiment 5: effects of acute administration of SNP and 8-OH-DPAT on rats’ performance in the motor activity test

**Fig. 4:**
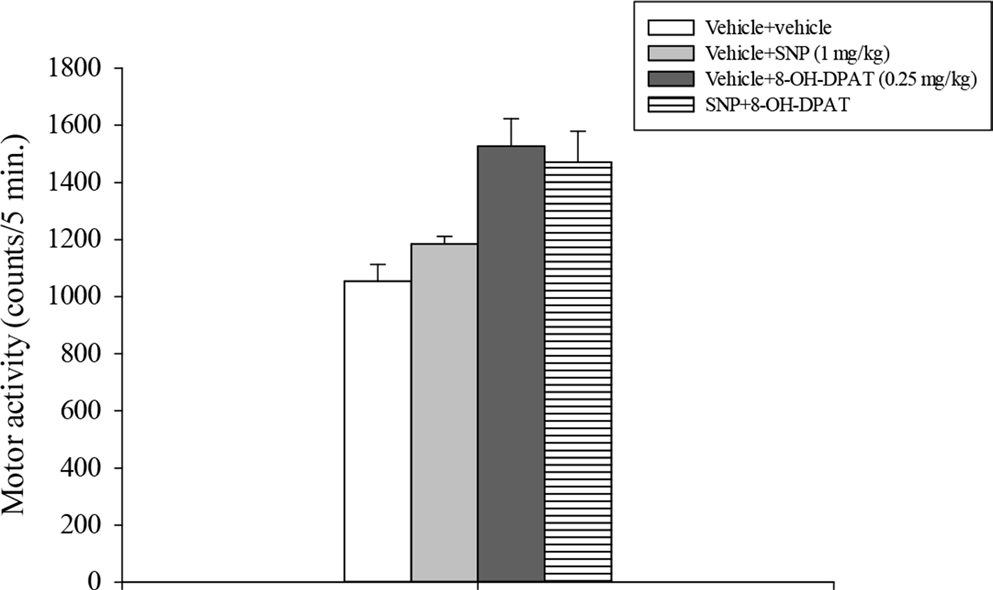
Locomotor activity test. SNP and 8-OH-DPAT were injected i.p. 30 and 10 min respectively before testing. The histogram shows the means ± SEM of 8 rats per treatment group

Motor activity data analysis showed a significant main effect of 8-OH-DPAT (*F*_1,31_ = 23.24, *p* < 0.001) but not of SNP or its interaction with 8-OH-DPAT (Fig. 4).

### Experiment 6: effects of acute administration of SNP and 8-OH-DPAT on rats’ performance in the OF test

Relative results are illustrated in Table 2. Regarding the number of squares crossed and the number of rearings data analysis revealed a statistically significant main effect of 8-OH-DPAT (*F*_1,31_ = 24.89, *p* < 0.001), for squares crossed and (*F*_1,31_ = 70.24, *p* < 0.001, for number of rearings). A significant main effect of SNP or a significant interaction of it with 8-OH-DPAT was not observed in the parameters above described. Concerning the amount of time spent by rats in the central area of the apparatus, a statistically significant interaction between 8-OH-DPAT and SNP was revealed (*F*_1,31_ = 7.23, *p* = 0.012). Moreover, a significant main effect either of 8-OH-DPAT (*F*_1,31_ = 31.81, *p* < 0.001) or of SNP (*F*_1,31_ = 5.49, *p* = 0.026) was evidenced.

**Table 2:** Effects of acute treatment with sodium nitroprusside (SNP) and 8-OH-DPAT on rats’ performance in the open field test

TREATMENT	Number of squares crossed	Number of rearings	Time spent in the central zone (s.)
Vehicle + vehicle	78.6 ± 5.9	30.5 ± 1.9	5.8 ± 1
SNP (1 mg/kg) + vehicle	92.3 ± 5.4	35.6 ± 3.4	20.9 ± 2.1*
8-OH-DPAT (0.25 mg/kg) + vehicle	123.3 ± 10.4	5.1 ± 1.9	5.5 ± 1.9
8-OH-DPAT + SNP	125.3 ± 8.4	9.9 ± 4.3	7.6 ± 1.3

SNP and 8-OH-DPAT were injected i.p. 30–10 min before testing respectively

The values are mean ± SEM (*n* = 8 rats per group). *p < 0.05 vs. all the other groups

Post-hoc analysis carried out showed that animals that have received vehicle + SNP spent more time in the central zone of the open field arena as compared to all the other counterparts (*p* < 0.05).

## Discussion

Accordingly, to prior results (Orfanidou et al. 2017; Papageorgoulis et al. 2020; Peristeri and Pitsikas 2022) treatment with 1 mg/kg SNP induced a clear anxiolytic effect in rats evidenced both in the L/D box and the OF tests. Specifically, in all the distinct experiments of the present study, SNP dramatically increased the amount of time spent by rats either in the lit compartment of the L/D box or in the central area of the OF arena in comparison to all the other experimental groups.

Flumazenil, a GABA_A_-benzodiazepine receptor antagonist, eradicated the above- described anti-anxiety-like action of SNP. Flumazenil alone did not affect rats’ performance either in the L/D or in the OF test. This set of findings indicates that the GABA_A_-benzodiazepine receptor binding site might be a target of the anxiolytic effects exerted by SNP. The abolishment of the anti-anxiety-like effect of SNP by flumazenil supports the theory that SNP may act either directly or indirectly on the GABA_A_-benzodiazepine receptor. A direct interaction of SNP with the GABA_A_-benzodiazepine receptor could implicate SNP’s action on Cl^−^ conductance. An indirect action of SNP might depend on the regulation of metabolism and/or release of GABA. In this regard, it has been reported that SNP, in a dose-dependent manner, stimulate GABA efflux from various brain structures (Prast and Philippu 2001), directly activate GABA_A_ receptor function (Castel and Vaudry 2001) and strengthen inhibitory GABAergic transmission (Larson et al. 2020). Finally, reduced NOergic activity is associated with a decrement of the anxiolytic effects of benzodiazepines (Elfline et al. 2004).

The 5-HT_1A_ receptor agonist 8-OH-DPAT by reducing the time spent by rats either in the light chamber of the L/D box or in the central zone of the OF arena abolished the anxiolytic effects of SNP. These findings propose that the anti-anxiety-like action of SNP might be mediated through its interplay with the 5-H_1A_ receptor.

In agreement with previous research (Arrant et al. 2013; Rombola’ et al. 2020) 8-OH-DPAT exhibited an anxiogenic profile in the L/D box and OF tests. Specifically, it decreased the first latency to enter the dark compartment, the number of transitions between the two chambers of the apparatus and the time spent in the aversive lit compartment of the L/D box. Concerning the OF test, 8-OH-DPAT dramatically reduced the time consumed by rats in the central area of the OF arena. Finally, in line with prior studies (Bjork et al. 1992; Nicolaus et al. 2023) 8-OH-DPAT diminished the number of rearing episodes. Interestingly, it has been suggested that reduction of rearing activity correlates with an increase of anxiety (Imre et al. 2006). Conforming to prior reports (Bjork et al. 1992; Kalkman et al. 1989, Müller et al. 2003), treatment with 8-OH-DPAT caused hypermotility since it increased rats’ motor activity and the number of squares crossed.

It has been previously reported that post-synaptic microinjection of 8-OH-DPAT into the hippocampus, medial septum, and basolateral amygdala caused anxiogenesis. On the contrary, pre-synaptic infusion of 8-OH-DPAT into the median and dorsal raphe induced an anxiolytic effect in rodents (De Almeida et al. 1998; Engin and Treit 2008; File et al. 1996). Therefore, the anxiogenic effects of 8-OH-DPAT shown in this study may be mediated preferably by the post-synaptic 5-HT_1A_ receptor. Based on the above, it can be hypothesized that the site of interaction between SNP and 8-OH-DPAT may be the post-synaptic 5-HT_1A_ receptor. The well-documented co-existence of nNOS-containing neurons and serotonin in hippocampus, medial septum and amygdala, which are brain areas rich of 5-HT_1A_ receptors and critically involved in anxiety (Bredt et al. 1991; Dawson et al. 1991) along with the ability of NO donors to modulate serotonin release from the above cited areas might be a support of this hypothesis (Prast and Philippu 2001). Additional research, however, is required aiming to elucidate this topic.

It has been shown that both SNP and flumazenil did not affect parameters reflecting motility (e.g., general activity, the number of transitions between the two chambers in the L/D test and the number of squares crossed and rearings in the OF test). The latter indicates that the implication of motor factors in the effects exerted by these molecules on variables evaluating anxiety seems unlikely. In the same vein, the anxiogenic effects expressed by 8-OH-DPAT cannot be associated with a general motor depression since this 5-HT_1A_ receptor agonist increased motor activity.

It is important to emphasize that the present study was performed exclusively with male rats, which limits the aim and general applicability of the current results. It is firmly established that anxiety occurs mostly in females than males (Kessler et al. 2012). Further research, thus, is required by using both male and female rodents aiming to definitively establish the validity of the present findings. Supplementary experiments (e.g., neurochemical, electrophysiological and molecular) are also needed aiming to reinforce the quality of the here reported behavioural results.

In summary, the present results indicate that a functional interaction between SNP with the GABAergic and the serotonergic system might underlie its anti-anxiety-like effects.

## Data Availability

The data that support the findings of this study are available upon request from the corresponding author.
